# Supplementary material for: Stem Cells Derived from Human Exfoliated Deciduous Teeth Functional Assessment: Exploring the Changes of Free Fatty Acids Composition during Cultivation
Source: Int J Mol Sci. 2023 Dec 8;24(24):17249. doi: 10.3390/ijms242417249 (PMC10743411; doi:10.3390/ijms242417249)
Supplement: Supplementary file 1 [file ijms-24-17249-s001.zip › ijms-2729559-supplementary.pdf]

Supplementary Materials

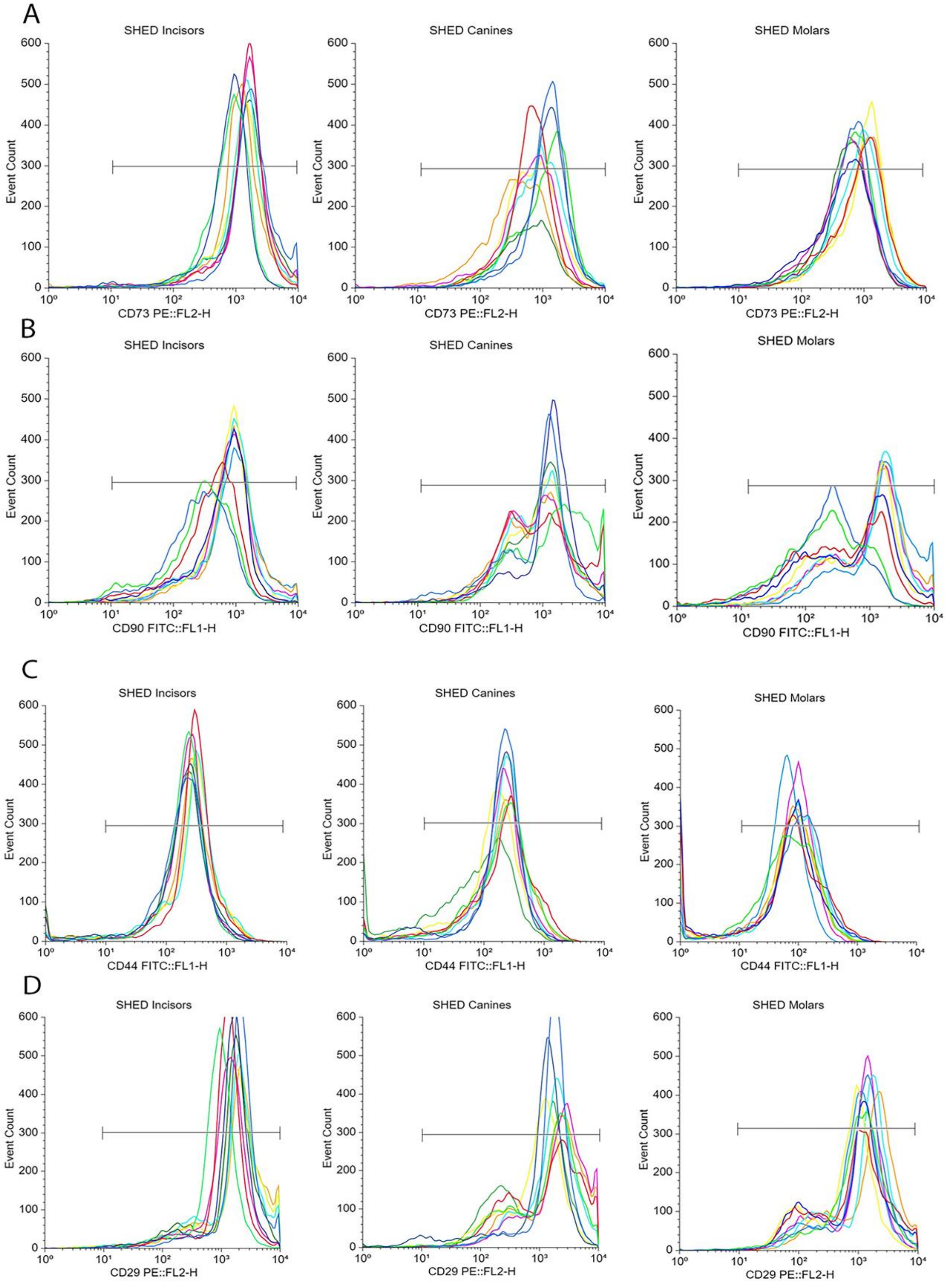

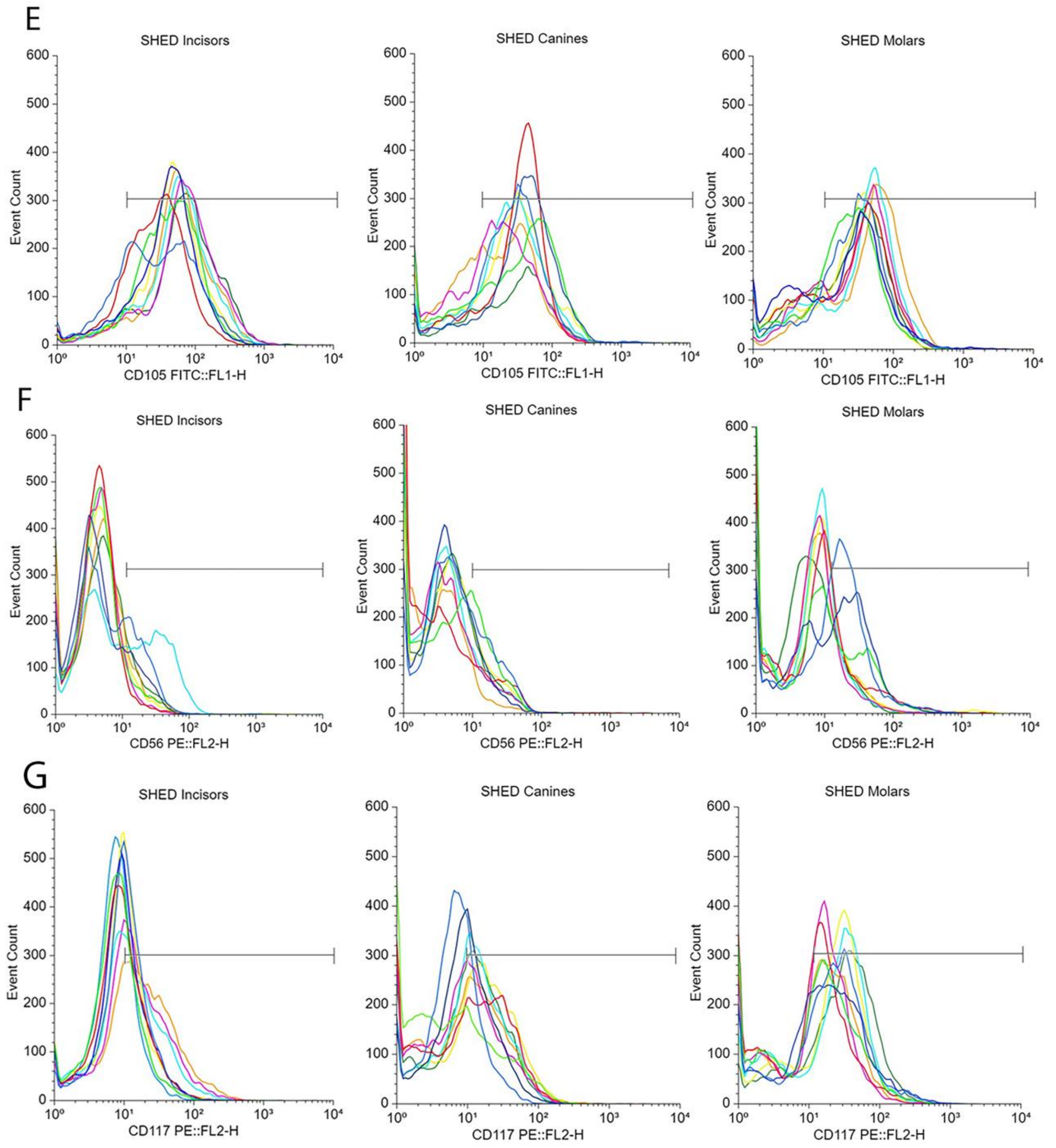

Figure S1. Representative histograms illustrating the phenotypic evaluation of SHED populations by flow cytometry from passage 2 to 10. Fig 1.A presents histograms for the CD73 marker in SHED populations from incisors, canines, and molars at passages 2 to 10. In Fig 1.B, histograms display the CD90 marker in SHED populations of incisors, canines, and molars across passages 2 to 10. Similarly, Fig 1.C showcases histograms for the CD44 marker in SHED populations from incisors, canines, and molars at passages 2 to 10. Fig 1.D and Fig 1.E provide representative histograms for the CD44 and CD105 markers, respectively, in SHED populations from incisors, canines, and molars at passages 2 to 10. Finally, Fig 1.F and Fig 1.G

depict histograms for the CD56 and CD117 markers, respectively, in SHED populations from incisors, canines, and molars at passages 2 to 10.

Common Name	Corr Area	% of Total
Lauric acid	1803903	2.50%
Myristic acid	1812212	2.51%
Palmitic acid	50515264	70.04%
Elaidic acid	8721937	12.09%
Stearic acid	9266487	12.85%

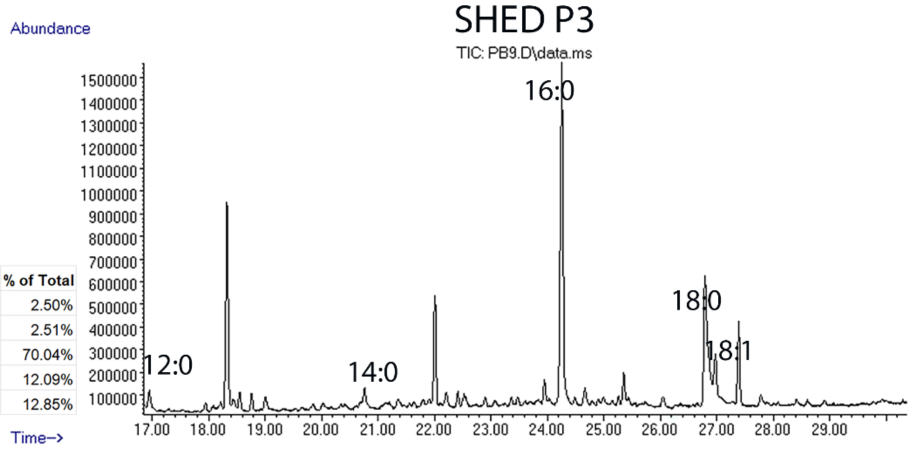

Common Name	Corr Area	% of Total
Lauric acid	4157905	3.35%
Myristic acid	4287026	3.45%
Palmitic acid	89460754	72.06%
Elaidic aicd	11316697	9.12%
Stearic acid	14181146	11.42%

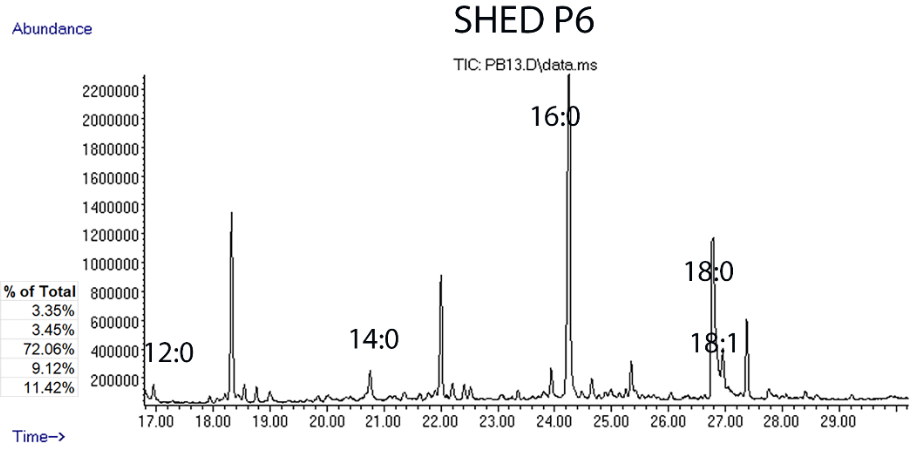

Common Name	Corr Area	% of Total
Lauric acid	1298561	0.99%
Myristic acid	2815338	2.14%
Palmitic acid	82171880	62.45%
Oleic acid	16349106	12.43%
Elaidic acid	4120090	3.13%
Stearic acid	21057870	16.00%
Arachidonic acid	3772550	2.87%

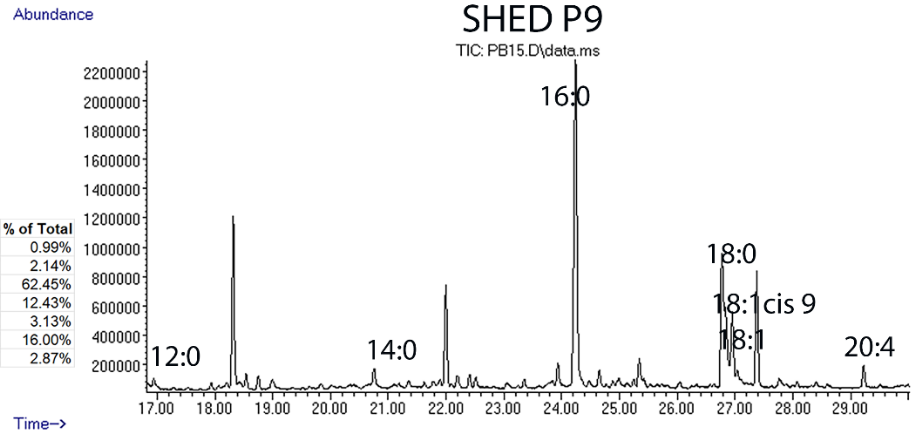

Figure S2. Representative chromatograms presenting the fatty acid composition assessed by GC-MS. The fatty acid content of SHED populations was analyzed at different passages (P3, P6, and P9) during their cultivation using GS-MS
